# Discovery of novel multidrug resistance protein 4 (MRP4) inhibitors as active agents reducing resistance to anticancer drug 6-Mercaptopurine (6-MP) by structure and ligand-based virtual screening

**DOI:** 10.1371/journal.pone.0205175

**Published:** 2018-10-15

**Authors:** Ya Chen, Xia Yuan, Zhangping Xiao, Hongwei Jin, Liangren Zhang, Zhenming Liu

**Affiliations:** State Key Laboratory of Natural and Biomimetic Drugs, School of Pharmaceutical Sciences, Peking University, Beijing, P. R. China; Columbia University, UNITED STATES

## Abstract

Multidrug resistance protein 4 (MRP4/ABCC4) is an ATP-binding cassette (ABC) transporter. It is associated with multidrug resistance (MDR), which is becoming a growing challenge to the treatment of cancer and infections. In the context of several types of cancer in which MRP4 is overexpressed, MRP4 inhibition manifests striking effects against cancer progression and drug resistance. In this study, we combined ligand-based and structure-based drug design strategy, by searching the SPECS chemical library to find compounds that are most likely to bind to MRP4. Clustering analysis based on a two-dimensional fingerprint was performed to help with visual selection of potential compounds. Cell viability assays with potential inhibitors and the anticancer drug 6-MP were carried out to identify their bioactivity. As a result, 39 compounds were tested and seven of them reached inhibition above 55% with 6-MP. Then compound **Cpd23** was discovered to improve HEK293/MRP4 cell sensibility to 6-MP dramatically, and low concentration **Cpd23** (5 μM) achieved the equivalent effect of 50 μM MK571. The accumulation of 6-MP was determined by validated high-performance liquid chromatography methods, and pretreatment of the HEK293/MRP4 cells with 50 μM MK571 or **Cpd23** resulted in significantly increased accumulation of 6-MP by approximately 1.5 times. This compound was first reported with a novel scaffold compared with previously known MRP4 inhibitors, which is a hopeful molecular tool that can be used for overcoming multidrug resistance research.

## Introduction

In the treatment of cancer and infections, when cells are exposed to chemotherapeutic drugs and antibiotics, they can develop multidrug resistance (MDR). Several mechanisms contribute to MDR including efflux molecules outside of cells via drug transporters. To overcome MDR, exploring membrane transport-modulating agents (MTMA) of drug efflux transporters would be a supplementary therapy [1, 2].

Multidrug resistance protein 4 (MRP4/ABCC4), a protein consisting of 1,325 amino acids encoded by the ABCC4 gene, is an ATP-dependent transporter and its main function is pumping organic anions across biological membranes against a concentration gradient [3]. Among its endogenous substrates, most are signaling molecules (e.g., the eicosanoids prostaglandin E_2_, leukotriene B_4,_ and thromboxane TXB_2_) and second messengers (the cyclic nucleotides cAMP and cGMP), as well as bile acids, conjugated steroids, and folic acid [4, 5]. MRP4 also has the ability to efflux a range of therapeutic agents, particularly anticancer drugs, such as thiopurines, camptothecins, and methotrexate; nucleoside-based antivirals, including ganciclovir and nelfinavir; and cardiovascular therapeutics e.g. hydrochlorothiazide and furosemide [4–6]. Experimental studies have proved that MRP4 involved in resistance to anticancer agent topotecan, suggesting that MRP4 MTMA may improve the therapeutic efficacy of drugs that are MRP4 substrates [7].

MRP4 has the typical core structure of ABC transporters. It is composed of two transmembrane domains (TMDs), and two nucleotide binding domains (NBDs). Each TMD consists of six transmembrane helices (TMHs) that are important for ligand binding and NBDs bind and hydrolyze ATP to drive transport [8]. MRP4 is widely expressed in most human tissues, including brain, liver, kidney, pancreas, adrenal glands, erythrocytes, and platelets [3, 5]. Depending upon cell types, MRP4 can be located either apically or basolaterally [3, 5]. Because of its broad substrate specificity and localization, MRP4 plays a role in the disposition of various drugs and their metabolites. Thus MRP4 may play a key part in protecting cells and extracellular signal transduction pathways [5].

**Fig 1:**
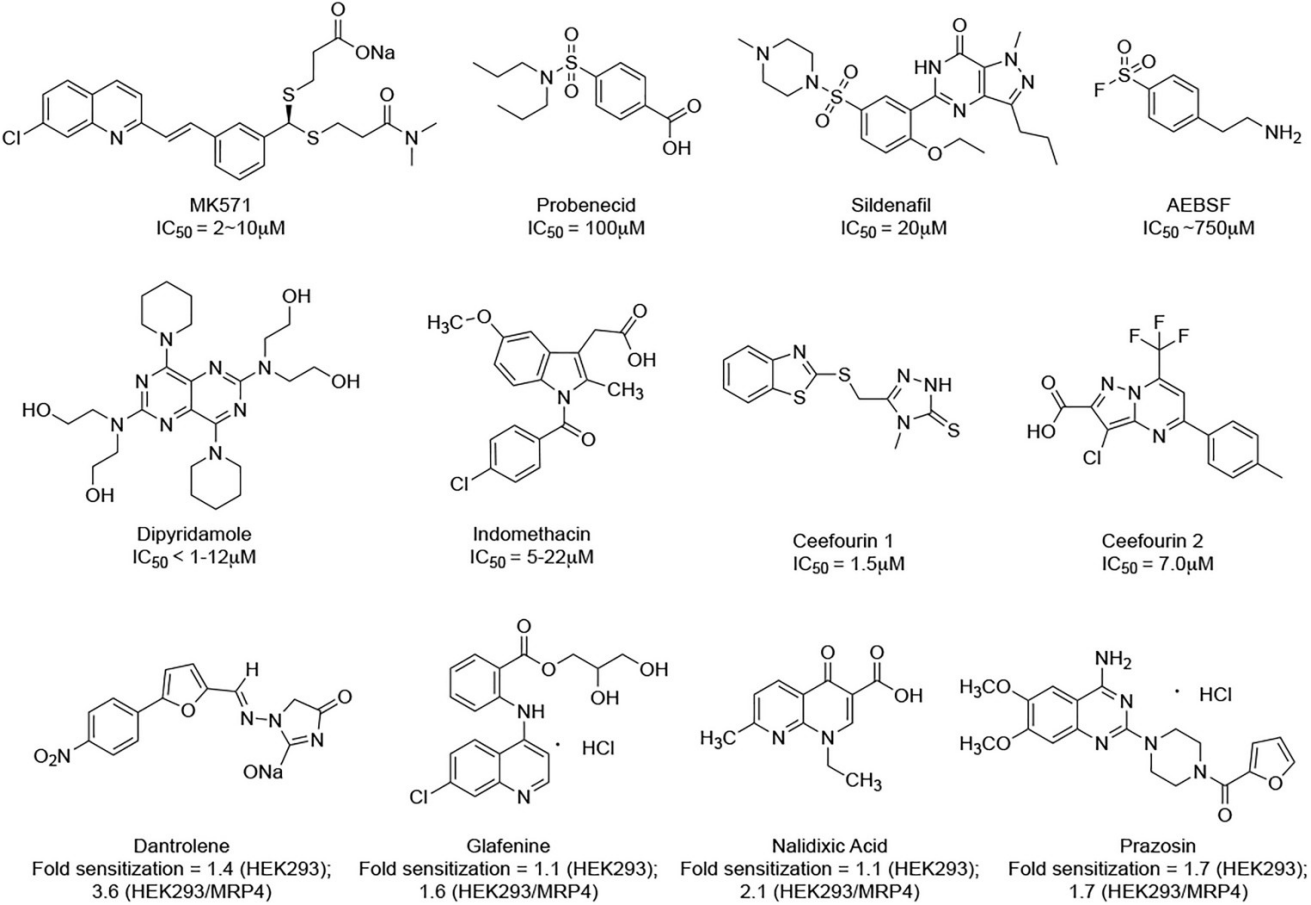
Known MRP4 inhibitors and their bioactivity. Fold sensitization = (IC_50_ cytotoxic + DMSO)/ (IC_50_ cytotoxic 6-MP + inhibitor).

Despite the interest in MRP4’s biological function, relatively few small-molecule inhibitors are available. The known inhibitors are generally with low potency and low specificity [5] (Fig 1). A clinically tested compound, MK571 ((*E*)-3-[[[3-[2-(7-chloro-2-quinolinyl)ethenyl]phenyl]-[[3-dimethylamino]-3-oxopropyl]thio]methyl]thio)-propanoic acid), is a widely used MRP4 inhibitor. However, MK571 also inhibits MRP1, MRP2, MRP3, MRP5, and phosphodiesterases [9–13]. In addition to probenecid, sidenafil, AEBSF, dipyridamole, and indomethacin, which are weak and non-selective MRP4 inhibitors [5], Cheung et al.[14] identified Ceefourin 1 and Ceefourin 2 as highly selective inhibitors of MRP4 by high-throughput screening (HTS) of a diverse small-molecule library. They also identified a range of previously unknown MRP4 inhibitors from a library of established drugs and well-characterized bioactive compounds [15]. Compared to HTS, virtual screening would be an efficient way to find more novel MRP4 inhibitors, and expand their structure-activity relationships.

The knowledge of MRP4’s structural, biological, and pharmacological properties is limited. Due to the lack of X-ray crystal structures, predicting MRP4’s structure by homology modeling might be an alternative way to gain structural insight into MRP4. The molecular properties of MRP4, especially the substrate translocation pathway, could be effective tools to design therapeutic agents that reducing the consequences of MDR.

There are currently a number of crystal structures that can be used as templates for MRP4 modeling. For example, the crystal structure of bacterial ABC transporter *Sav*1866 from *Staphylococcus aureus* was used by Ravna and colleagues to construct a MRP4 model [16], which represents the outward-facing state of MRP4. They also built the inward-facing state of MRP4 using the X-ray crystal structure of *Escherichia coli MsbA* as the template [17]. Wittgen et al. built homology models of MRP4 in different states. They built the outward-facing model using a hybrid-template of the transmembrane domain of *Sav*1866 and the ATP-binding domain of human MRP1[18]. While for the inward-facing models, they used the X-ray structure of *Mus musculus* P-glycoprotein (P-gp) as the template [19]. As the development of structural biology, more and more ABC structures have been revealed [20–23], which provide more opportunities for structural modeling of MRP4. Our group has built three homology models of MRP4, which represent three key conformations of substrate transporting cycle [24].

At least two active sites of MRPs, the ATP binding site and substrate transport cavity, can be used as binding pockets of MRPs inhibitors. Sirisha and coworkers [25] presented molecular docking studies of a newly synthesized DHP derivative compound library to the crystal structure of MRP1-NBD1 and found two compounds that exhibit potent MRP1 inhibitory activity with IC_50_ values of 20 ± 4 μM and 14 ± 2 μM (mean ± SD), respectively. Prehm [26] identified a curcumin analogue as a hyaluronan export inhibitor by docking to the ATP binding site between NBD1 and NBD2 of MRP5. The superior hyaluronan export inhibitor prevented hyaluronan export from fibroblasts with an IC_50_ of 4.9 μM. Sager et al. [27] predicted several MRP5 inhibitors by virtual ligand screening (VLS) and using the large internal hypothetic drug binding cavity consisting of TMH1, TMH5, TMH6, TMH7, and TMH12 from MRP5. The two most potent inhibitors showed K_i_ of 50–100 nM. Herein, we employed the putative drug binding cavity in the substrate uptake cavity as the pocket to find MRP4 inhibitors.

The aim of this study was to find novel MRP4 inhibitors by computational guidance and test them for reducing resistance to the MRP4 substrate and anticancer drug 6-Mercaptopurine (6-MP). The method of ligand-based drug design, which is based on MK571, and structure-based drug design, which relies on the three-dimensional (3D) structure of MRP4, were combined to search for new MRP4 inhibitors in the SPECS database. Compounds from virtual screening were selected based on calculated binding affinity, probable hydrogen bond number, predicted water solubility, and clustering analysis. *In vitro* activity for MRP4 inhibition were tested combined with 6-MP.

## Materials and methods

### Three-dimensional similarity search

The SPECS database (http://www.specs.net/) was selected as the chemical library for virtual screening, and OpenEye software (OEChem version 1.9.1; OpenEye Scientific Software Inc.) was applied for the similarity search due to its fast and powerful implementation of three-dimensional (3D) shape in conformation generation and comparison [28, 29].

The compound database was prepared with an in-house protocol developed in Pipeline Pilot v7.5 (PP 7.5, Accelrys Software, Inc., San Diego, CA, USA.), in which the chemical structures formed 3D coordinates, were stripped of counter ions, minimized, and standardized. Then, the prepared database was filtered by BlockBuster filter in FILTER (version 2.2.1, OpenEye Scientific Software, Inc., Santa Fe, NM, USA) to remove molecules with unsatisfactory physicochemical properties with respect to molecular weight, number of heavy atoms, and solubility (see details in filter_blockbuster.txt of OpenEye software). After this, the database was processed with OMEGA (version 2.4.5, OpenEye Scientific Software, Inc., Santa Fe, NM, USA) to generate up to 500 conformations for each molecule. OMEGA was designed for computer-aided drug design with large libraries. It is very effective at reproducing bioactive conformations [30].

We employed ROCS [31] (Rapid Overlay of Chemical Structures, version 3.2.0, OpenEye Scientific Software, Inc., Santa Fe, NM, USA) for 3D shape comparison. Three conformers of MK571 served as initial conformations to generate ROCS queries: the first and second queries are different docking poses with the MRP4 model by AutoDock 4 [32], and the third query is the lowest-energy conformer generated by OMEGA. The top-ranked 10000 conformations with the highest Shape Tanimoto similarity values of each query were returned in rank order as hits. For electrostatic comparisons by EON 2.2.0, three conformers of MK571 were used again for comparison to re-rank conformations separately. Then conformations with EON_ShapeTanimoto similarity values above 0.7 (ranging from 0–1, 1 represents complete overlap [28, 29]) of each sets were kept.

### Docking-based virtual screening

The compounds from the 3D similarity search were docked into the MRP4 model by AutoDock Vina [33]. The docking pocket of MK571 was defined as the center of MRP4 “docking active site” with a 26 × 26 × 26 Å^3^ grid volume. We used the default parameters for the docking variables and the nine energetically most favorable binding poses were returned for each molecule. Gasteiger charges were calculated for the ligands and the receptor, and compounds were docked in their protonation state at pH 7.4. The pose with the best predicted binding affinity of each molecule was extracted and we also calculated the numbers of probable hydrogen bonds. The docking procedure was repeated three times. Water solubility at 25°C of each compound was calculated with the ADMET solubility prediction module of Discovery Studio 2.5 (Accelrys Software, Inc., San Diego, CA, USA). Molecules with ADMET solubility level in 0 (extremely low) and 1 (very low) were removed to ensure that the chosen compounds have acceptable solubility. The remaining compounds were clustered based on two-dimensional fingerprints, which are extended connectivity fingerprints of maximum diameter 6 (ECFP_6) fingerprint, and function class fingerprints of maximum diameter 6 (FCFP_6) fingerprint to assist the selection of compounds for experimental testing.

### Cell viability assays

Stable HEK293/MRP4 cells (purchased from the Netherland Cancer Institute) were seeded at 5×10^3^ cells per well into duplicate 96-well plates in 160 μl of DMEM/10% FBS and allowed to attach overnight. The following day, 20 μl of each compound in DMSO was transferred from a chemical library to a single well on each of the duplicate plates to give a final concentration of 10 μM for each in 0.1% DMSO. The positive control MK571 was at 50 μM, which is required to give strong inhibition of MRP4 using intact cells. Then, 20 μl of 6-MP in medium was added to one plate to give a final concentration of 15 μM. After 72 h incubation, cell viability was assessed using sulforhodamine B (SRB). We noted compounds that reduced cell viability to 45% or less in the presence of 6-MP (Inhibition_6-MP_ ≥55%). Hits were defined as those that met the criteria of reducing cell viability in the presence of 6-MP by at least 60% more than they did in the absence of 6-MP (Inhibition_6-MP_—Inhibition_untreated_ ≥60%), which means these compounds increase cell sensitive to 6-MP, but this effect are not because of their own toxicity. The IC_50_ of 6-MP in the presence of DMSO, MK571, or hit compounds was tested using HEK293 and HEK293/MRP4.

### Determination of 6-MP accumulation by HPLC

According to the determination method of CPT-11 and SN-38 [12], we revised the experiment to determine the accumulation of 6-MP under different conditions.

The accumulation of 6-MP in HEK293 and MRP4/HEK293 cells were examined in confluent cell cultures grown on 60-mm plastic culture dishes. Briefly, exponentially growing cells were exposed to 100 μM of 6-MP for 120 min at 37°C. The medium was aspirated off at the indicated times, and the dishes were rapidly rinsed five times with 5 ml of ice-cold PBS. HPLC analysis of the final washes guaranteed that they contained no residual 6-MP. After washing with ice-cold PBS, the cells were harvested and each cell pellet was suspended in 200 μl of extraction solution [acetonitrile/water (1:3, v/v)]. Then the mixture was sonicated, vortexed, and centrifuged. The supernatant was then injected into HPLC for concentration determination. Viable cells were monitored using the trypan blue exclusion method and the accumulation of 6-MP was expressed as nanograms per 10^6^ cells. Additionally, the effect of **Cpd23** (50 μM) and MK571 (50 μM) on 6-MP accumulation was investigated. Both **Cpd23** and MK571 were prepared by dissolving them in DMSO and diluting by PBS. The final concentration of DMSO was 1% (v/v). The two inhibitors at indicated concentrations showed little cytotoxicity (<5%) when incubated for 2 h. **Cpd23** or MK571 was preincubated with cells for 2 h. Subsequently, the cells were washed with warm PBS buffer for five times. After continued incubation for 2 h treated by 6-MP, the cells were washed five times with warm PBS. The cells were then harvested, lysed by sonication, and extracted using an ice-cold acetonitrile/water mixture (1:3, v/v). The supernatant was injected into HPLC for the determination of 6-MP.

Separations were performed on a 250 mm × 4.6 mm Venusil XBP C18 column at 25°C using an Agilent 1260 Infinity Liquid Chromatography. The flow rate was 1 ml/min with a mobile phase of acetonitrile and pure water. Elution was made with a gradient increasing acetonitrile in proportions of 2.5%, 4.0%, 8.0%, and 27.5% up to 90.0% (v/v) during 30 min. The strongest UV absorption of 6-MP was around 320 nm and there was hardly any absorption near 254 nm. So the absorption wavelength of 320 nm was chosen to determine the concentration. External standardization was adopted to quantify 6-MP in cytochylema, meanwhile we calibrated the results. A standard curve was drawn to show the area ratio between 6-MP and the external standard substance deoxyadenosine (dA) due to the increase of 6-MP. Analyses were made by injection of 15 μl of mixture of equal external standard dA and cytochylema to determine the absorption area.

## Results and discussion

### Virtual screening

**Fig 2:**
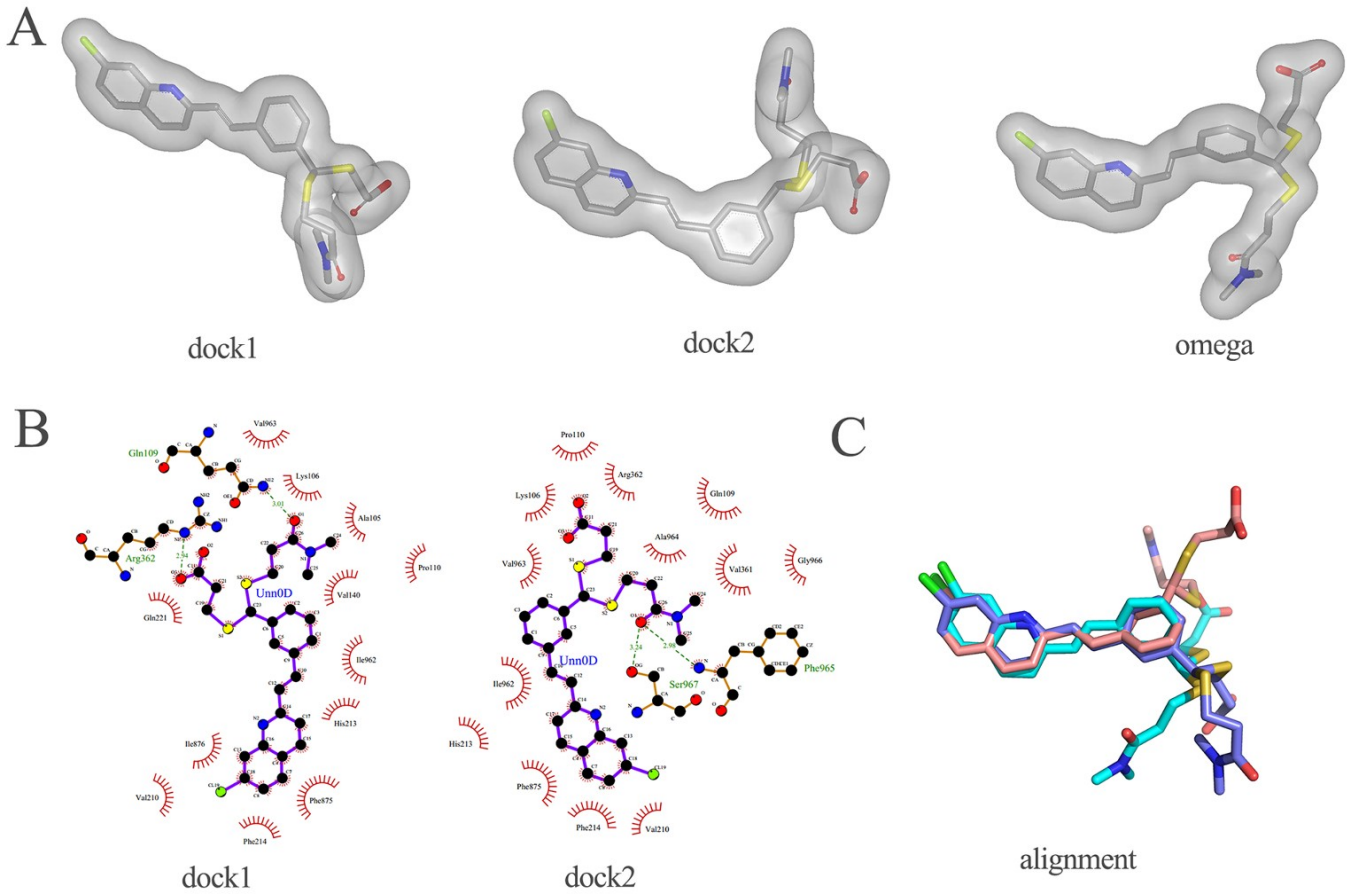
**A) Three queries used in 3D similarity search. B) Two different binding modes between MRP4 and MK571**. Created by LigPlot+ [34] to represent the interactions, showing the inhibitors (purple), residues involved in hydrogen bonding with the ligand (brown), along with their hydrogen bonds (green), and residues involved in non-bonded interactions (red spikes). P-gp equivalent residues showed in black box. **C) Alignment of the three queries**. Carbon atoms in dock1, dock2, and omega are in slate, salmon, and cyan, respectively.

Three different conformers of MK571 (Fig 2A) were used as queries in the 3D similarity search. MK571 is a known inhibitor of MRP4. Although its binding sites have not been identified yet, it is likely that MK571 shares a common location with MRP4 substrates. So we defined key residues in substrate transport as the active site. Dock1 and dock2 were two different docking poses of MK571 against the MRP4 model, while omega was the lowest-energy conformer generated by OMEGA.

**Fig 3:**
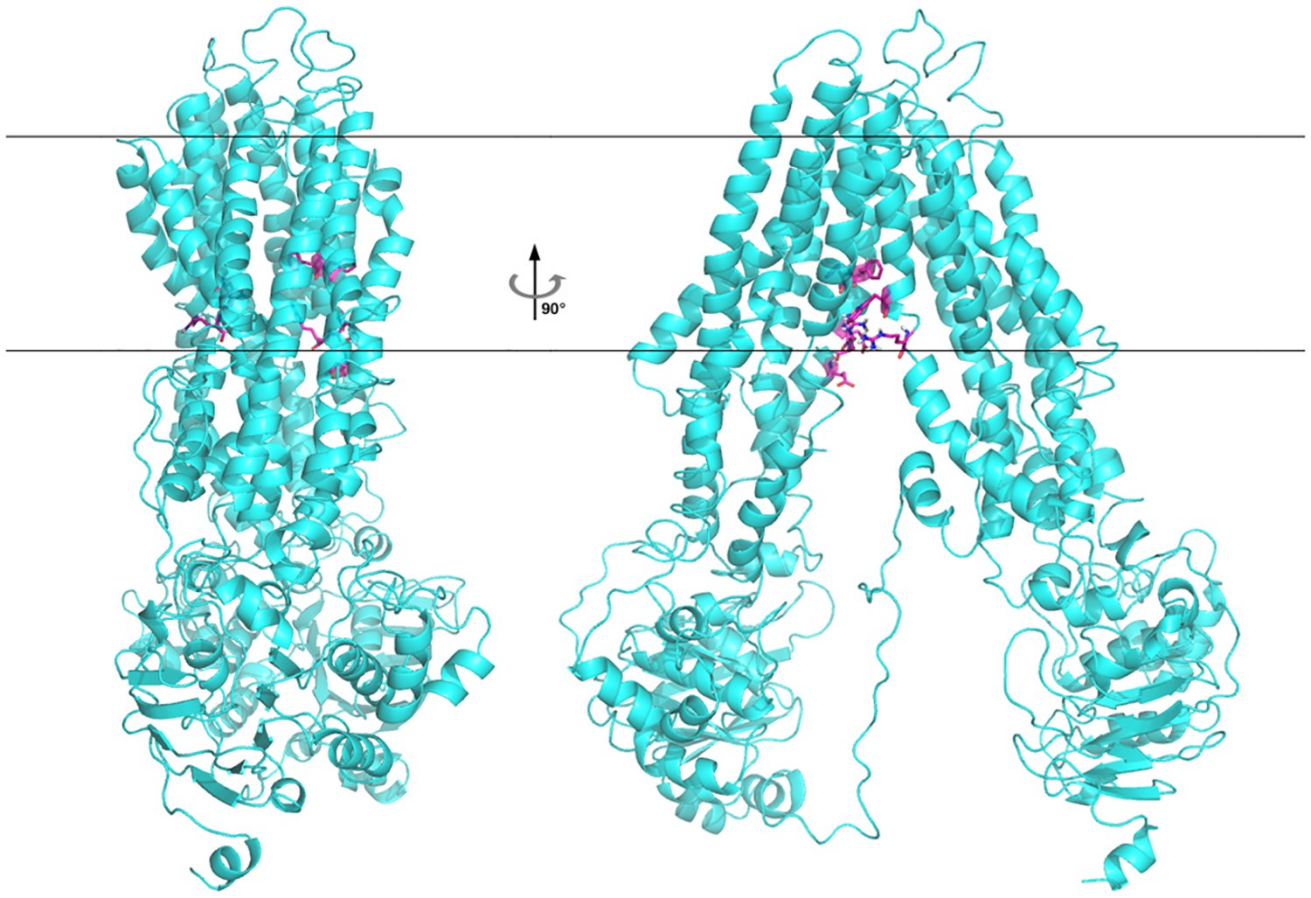
The homology model of MRP4, seen from two different views rotated 90° perpendicular to the membrane. Phe368, Phe369, Glu374, Arg375, and Glu378 in TMH6, and Trp995 and Arg998 in TMH12 of MRP4, which are important residues for substrates binding were shown in magenta.

We used the MRP4 model from homology modeling based on the *Caenorhabditis elegans* P-gp and NBD1 of human MPR1 (shown in Fig 3) for molecular docking. It is in an inward-facing conformation with the NBDs wide separated. This conformation is regarded as the initial state of substrate transport, thus it would be more important to discover active compounds against this conformation of MRP4. A large internal cavity open to the cytoplasm was formed by two transmembrane helix bundles: TMH1, 2, 3, 6, 10, 11 and TMH4, 5, 7, 8, 9, 12.

In a previous study, El-Sheikh et al. [18] revealed that Phe368, Phe369, Glu374, Arg375, and Glu378 in TMH6, as well as Arg998 in TMH12 of MRP4 were important for the transport function of MRP4. Wittgen and colleagues [19] investigated the effect of Phe368 (TMH6), Trp995, and Arg998 (TMH12) on the substrate-dependent transport activity of MRP4 and revealed that Arg998 seemed to be essential for the transport of all tested substrates. These amino acids were located in the large internal putative substrate binding cavity, such as Phe368, was located opposite to Trp995. The loop connecting NBD1 and TMD2 in the model featured a long loop with a short α-helix. There was no corresponding structure of this loop for modeling, so its structure was uncertain. This loop is also far from the binding pocket, and accordingly, it was not necessary for the purpose of this study.

As reported by site-directed mutagenesis studies on a homologue structure of MRP4 (P-gp) [35], the probable residues in a drug-binding site have corresponding residues in MRP4, which are “Glu103 (TMH1), Ser328 (TMH5), Gly359 (TMH6), Arg362 (TMH6), Val726 (TMH7), and Leu987 (TMH12)” [16]. These residues were located above the residues showed in Fig 3. We defined a larger binding pocket that covered two parts of these residues. The two docking poses of MK571 show different interactions with MRP4 (Fig 2B). Dock1 was bound into the pocket with an overall calculated binding affinity of -11.15 kcal/mol, while the binding mode of dock2 had an overall predicted binding affinity of -10.63 kcal/mol. The carbonyl group in dock1 might form a hydrogen bond with Gln109, and the carboxyl group was found to be engaged in hydrogen bonding with the positively charged side chain of Arg362. There were also hydrophobic interactions with other residues, such as Gln221, Ile876, Phe875, and Ile962. Dock2 had a different binding mode. The carbonyl group of dock2 was involved in hydrogen bonding with Phe965 and Ser967, and other hydrophobic interactions existed between dock2 and MRP4 as well. Arg362 was also a corresponding key residue in P-gp, and it had interactions with both poses. Unfortunately, residues which are also supposed to be important for MRP4 substrate transport such as Arg998 showed no interactions in both docking poses. The previous study did not test MK571 as a substrate, so MK571 might have a different binding mode comparing to other substrates of MRP4.

The three queries had similar poses when aligned using the substructure of 2-styrylquinoline, but the two side chains were in diverse conformations (Fig 2C). Root-mean-square deviations (RMSDs) are 7.0, 10.7, and 7.0 Angstrom for conformations between omega and dock1, omega and dock2, dock1 and dock2, respectively. We used all three queries to perform the 3D similarity search to include the active conformations.

**Fig 4:**
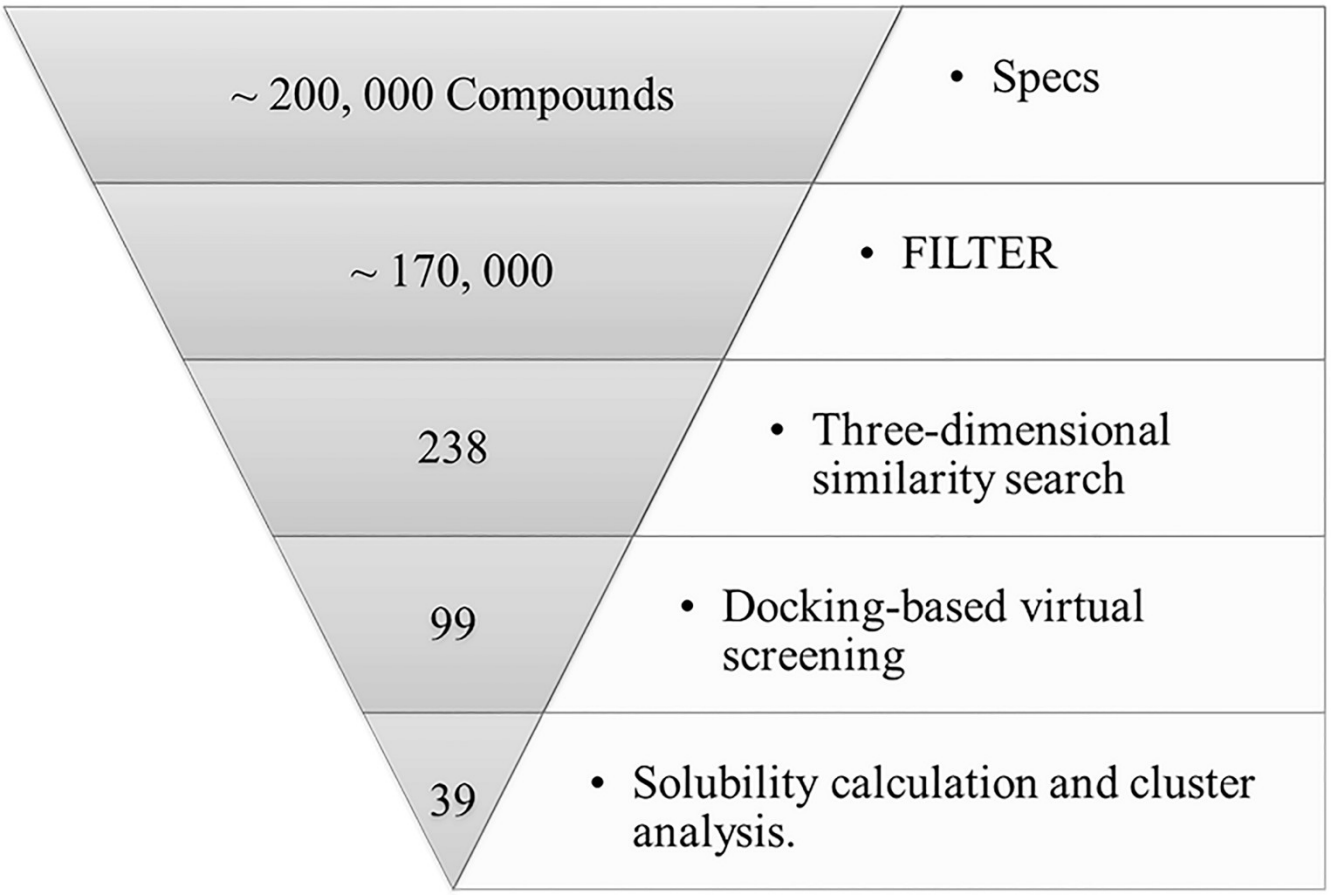
Schema for virtual screening strategy showing the number of compounds at each stage.

There were over 200,000 compounds for virtual screening in this study. A schema for the virtual screening strategy is shown in Fig 4. The prepared database was filtered with a basic standard in FILTER to remove molecules with unsatisfactory properties. After that, we obtained about 170,000 compounds for the similarity search.

After the 3D similarity search by ROCS and EON, dock1 found 160 compounds with similarity values above 0.7, while dock2 and omega found 128 and 40 molecules, respectively. Then the duplicate molecules were merged and returned 238 unique compounds in total. All 238 compounds were docked to the MRP4 model for three times, and conformers with the best predicted binding affinity were extracted for each molecule. Compounds 1) with a calculated binding affinity of no less than -9.0 kcal/mol in two or three binding times; 2) that failed to be observed forming hydrogen bonds with the MRP4 model in two or three times; or 3) with an average number of formed hydrogen bonds less than one, were discarded. A total of 99 compounds were obtained (Shape Tanimoto similarity values to three conformations of MK571 and binding affinities, number of probable hydrogen bond to the receptor for all 99 compounds were listed in S1 Table). Then an ADMET aqueous solubility properties calculation [36] was performed. ADMET solubility calculates the water solubility at 25°C, and ADMET solubility level ranks the solubility values into different classes: integral number 0–5 means extremely low, very low, low, good, optimal, and very soluble, respectively. We removed molecules with ADMET solubility levels in 0 (extremely low) and 1 (very low) to ensure that the selected compounds have acceptable water solubility. This step cut down the number of candidates to 65. Then we chose compounds by visual inspection with the assistance of cluster analysis by ECFP_6 and FCFP_6 fingerprints to keep molecules with diverse structures. Finally, 39 compounds were purchased from SPECS Corp (The Netherlands) for bioassays.

### Bioassays

**Fig 5:**
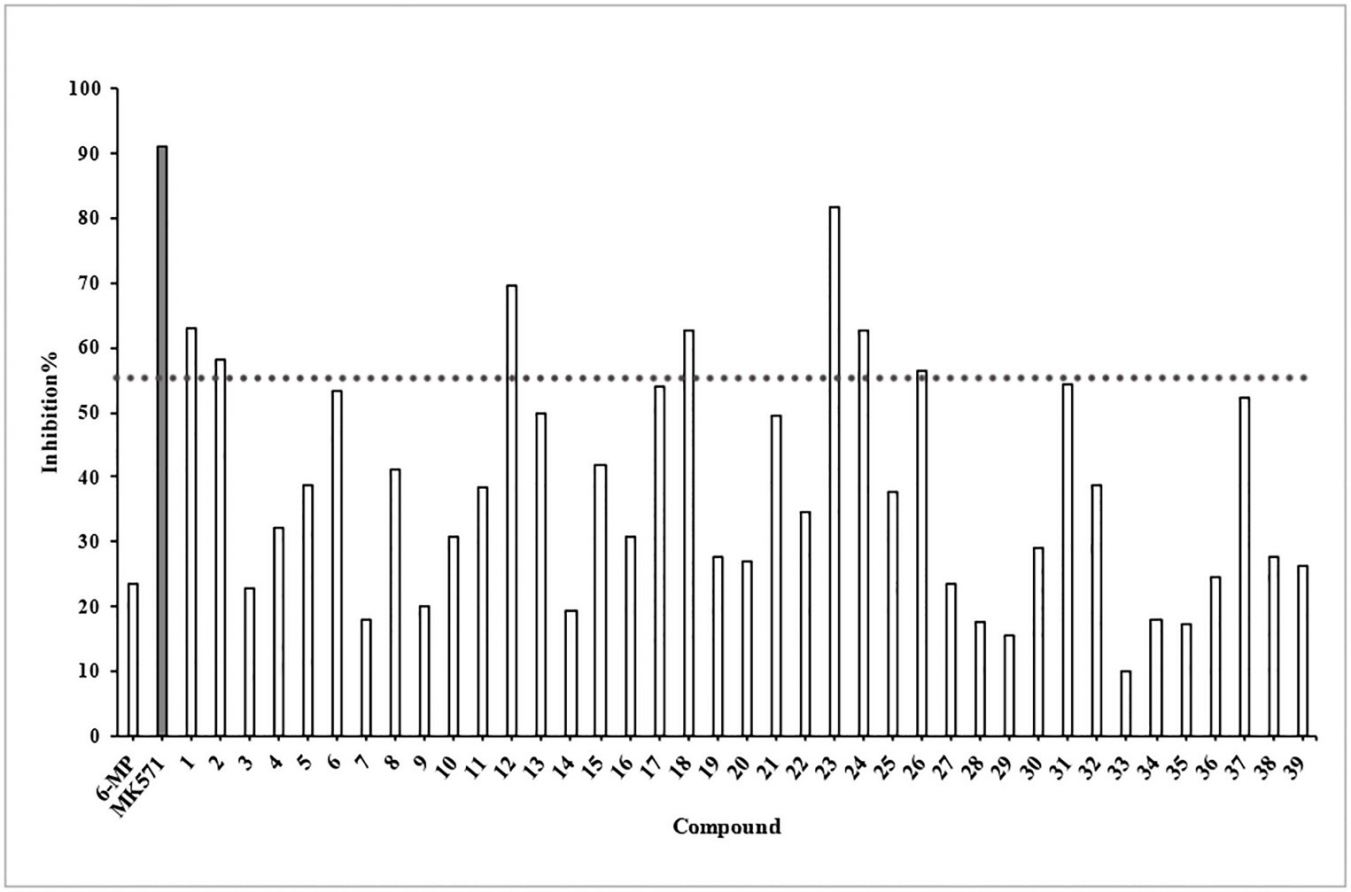
Cell inhibition rates of 39 selected compounds with 6-MP in stable HEK293/MRP4 cells. MK571 is the positive control. The dash line indicated the inhibition of 55%.

The primary screen measured sensitization of HEK293/MRP4 cells to the MRP4 substrate 6-MP. MRP4 was overexpressed in the HEK293/MRP4 cell line. We tested the 39 compounds at the concentration of 10 μM in the presence of 6-MP, using MK571 as the positive control. The inhibition rates are shown in Fig 5. The inhibition of 6-MP was only 23.48% at the concentration of 10 μM in HEK293/MRP4 and increased to above 90% with 50 μM of MK571. Only seven of the 39 molecules reached the inhibition above 55% with 6-MP.

**Fig 6:**
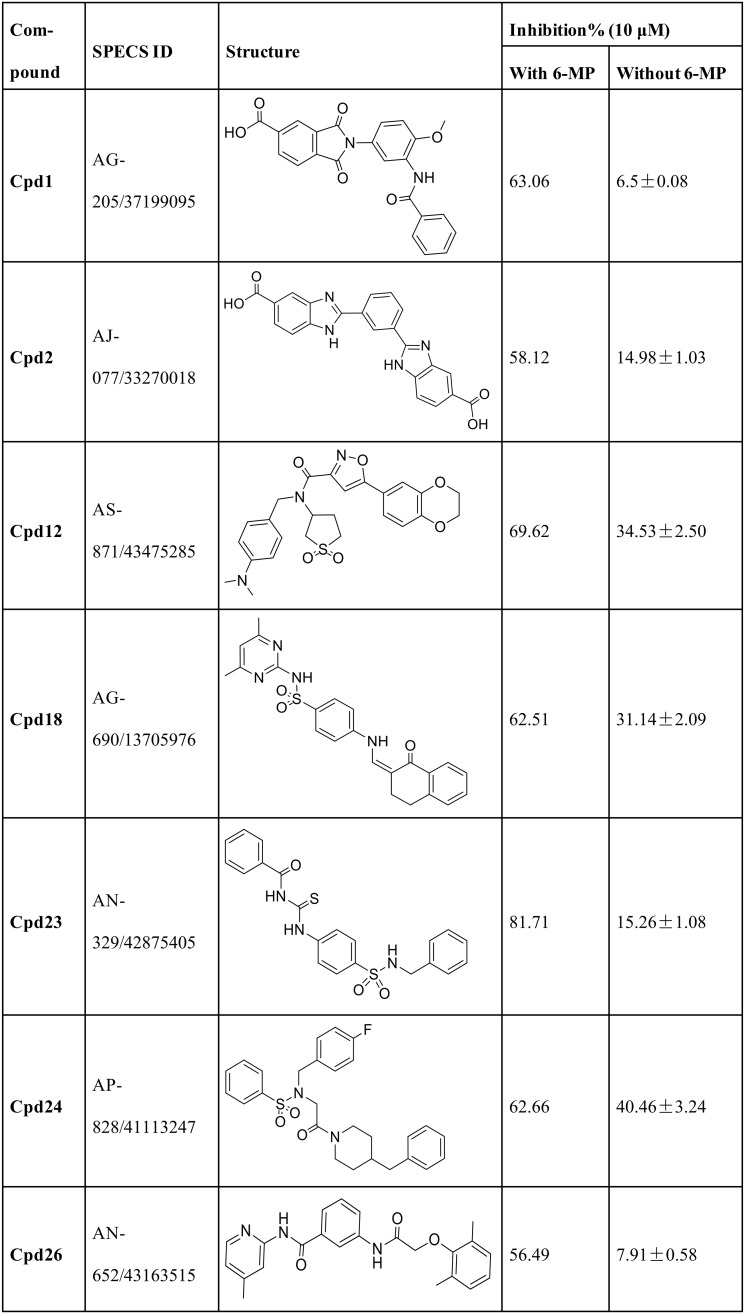
Compounds with the inhibition rates above 55% with 6-MP and their inhibition on the viability of HEK293/MRP4 without 6-MP.

We picked these seven compounds (calculated binding affinity results with SMILES format are provided in S2 Table) to test their own inhibition to HEK293/MRP4. Their structures and inhibition of the viability of HEK293/MRP4 with and without 6-MP are shown in Fig 6. **Cpd12**, **Cpd18**, and **Cpd24** showed inhibition between 30% to just above 40% without 6-MP, which means they have cell toxicity on their own, which contribute to the high inhibition rates while combing with 6-MP. **Cpd23** showed the highest activity with an inhibition rate of 81.71% at the concentration of 10 μM with 6-MP and only 15.26% without 6-MP. **Cpd1**, **Cpd2**, and **Cpd26** also had low inhibition rates on their own but did not reach inhibition rates as high as **Cpd23** did when combined with 6-MP. All seven compounds were subjected to the pan assay interference compounds (PAINS) online filter (http://cbligand.org/PAINS/) [37]. PAINS analysis showed that five of the seven compounds passed the filter which means they contain no substructure of PAINS; the exceptions were **Cpd1** and **Cpd12**. So we chose **Cpd23** for further study because it increased cell sensitivity to 6-MP, but not toxic on its own.

**Fig 7:**
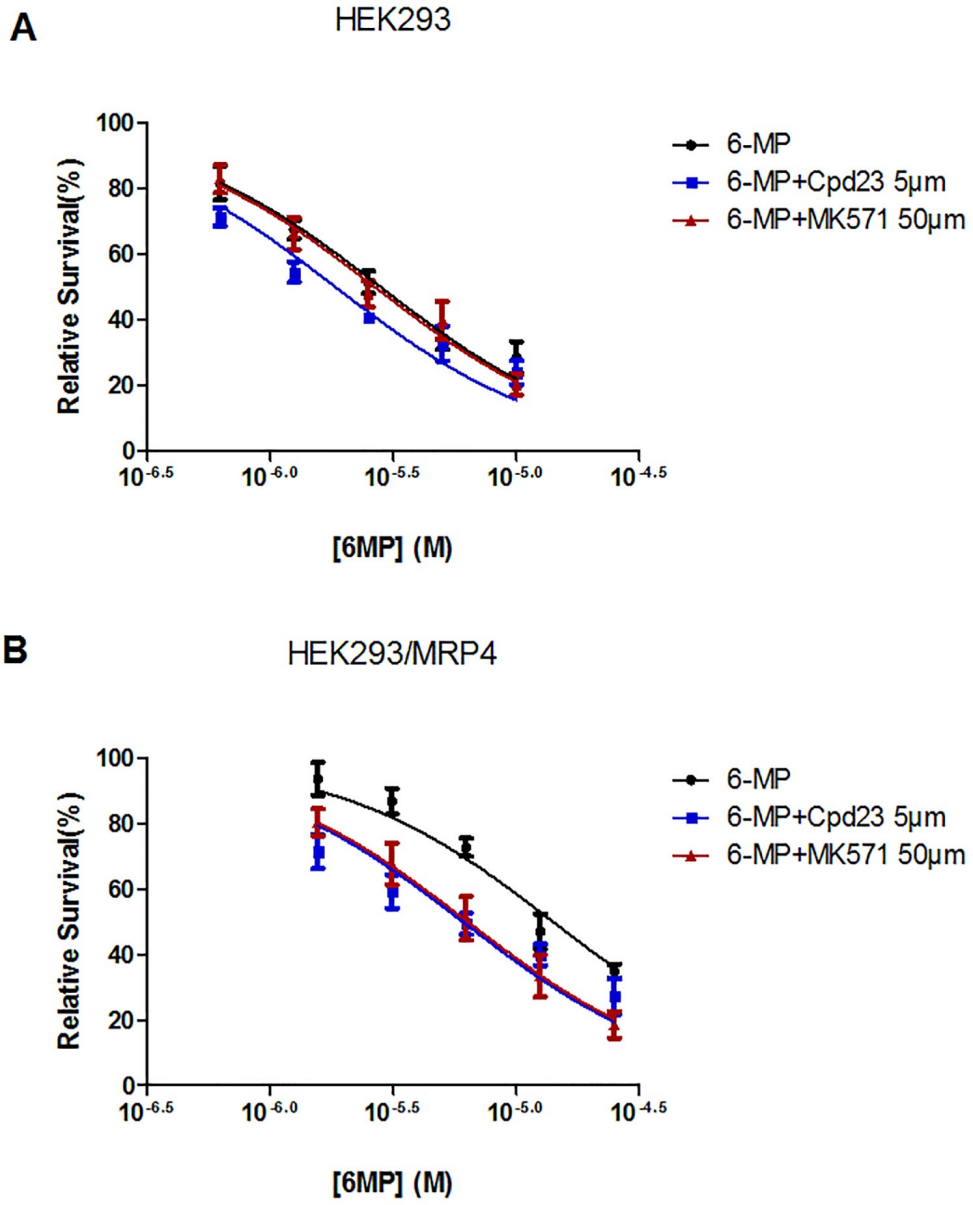
Cpd23 reverses 6-MP resistance conferred by MRP4. Dose-response curves for 6-MP on A) HEK293 and B) HEK293/MRP4 cells in the presence of DMSO, MK571, or **Cpd23**.

6-MP had IC_50_ of 2.81±0.35 μM on HEK293, while only 14.11±0.32 μM on MRP4 overexpressing cells (Table 1), indicating that MRP4 stably transfected HEK293 cells are resistant to 6-MP. At 5 μM, **Cpd23** had little effect on the 6-MP sensitivity of HEK293 cells (Table 1 and Fig 7A). In contrast, in combination with **Cpd23**, the IC_50_ of 6-MP substantially decreased in MRP4 overexpressing cells and the dose-response curve shifted to the left (Fig 7B). Comparable results were obtained for the positive control (50 μM of MK571) in each cell line. IC_50_ values are summarized in Table 1. A low concentration of **Cpd23** (5 μM) could achieve an equivalent effect of 50 μM MK571. Moreover, **Cpd23** had low inhibition in stable HEK293/MRP4 at 10 μM (15.26%) and did not have cell toxicity at the concentration of 5 μM (0.76%) (S1 Fig), thus the dose-response changes are possibly the results of synergism. The combination index (CI) of Cpd23 and 6-MP was calculated to estimate the synergism. The calculation formula is CI = C_A_/C_X,A_ + C_B_/C_X,B_ (C_A_ and C_B_ are the concentrations at which x% inhibition is achieved when A and B are combined; C_X,A_ and C_X,B_ are the concentrations at which x% inhibition is achieved when they used alone). When CI >1, CI = 1 or CI <1, the combined drugs have antagonism, addictive effect or synergism, individually. The Fa-CI plot shows CI <1 when using Cpd23 and 6-MP on HEK293/MRP4 cells (S1 Fig), which indicates their synergism.

**Table 1:** IC_50_ for 6-MP on HEK293 and HEK293/MRP4 cells independently or in the presence of MRP4 inhibitors.

Compounds	IC_50_ (μM)	
	HEK293	HEK293/MRP4
**6-MP + DMSOc**	2.81±0.35	14.11±0.32
**6-MP + MK571 (50 μM)**	2.66±0.28	6.35±0.68
**6-MP + Cpd23 (5 μM)**	1.84±0.10	6.06±0.81

**Fig 8:**
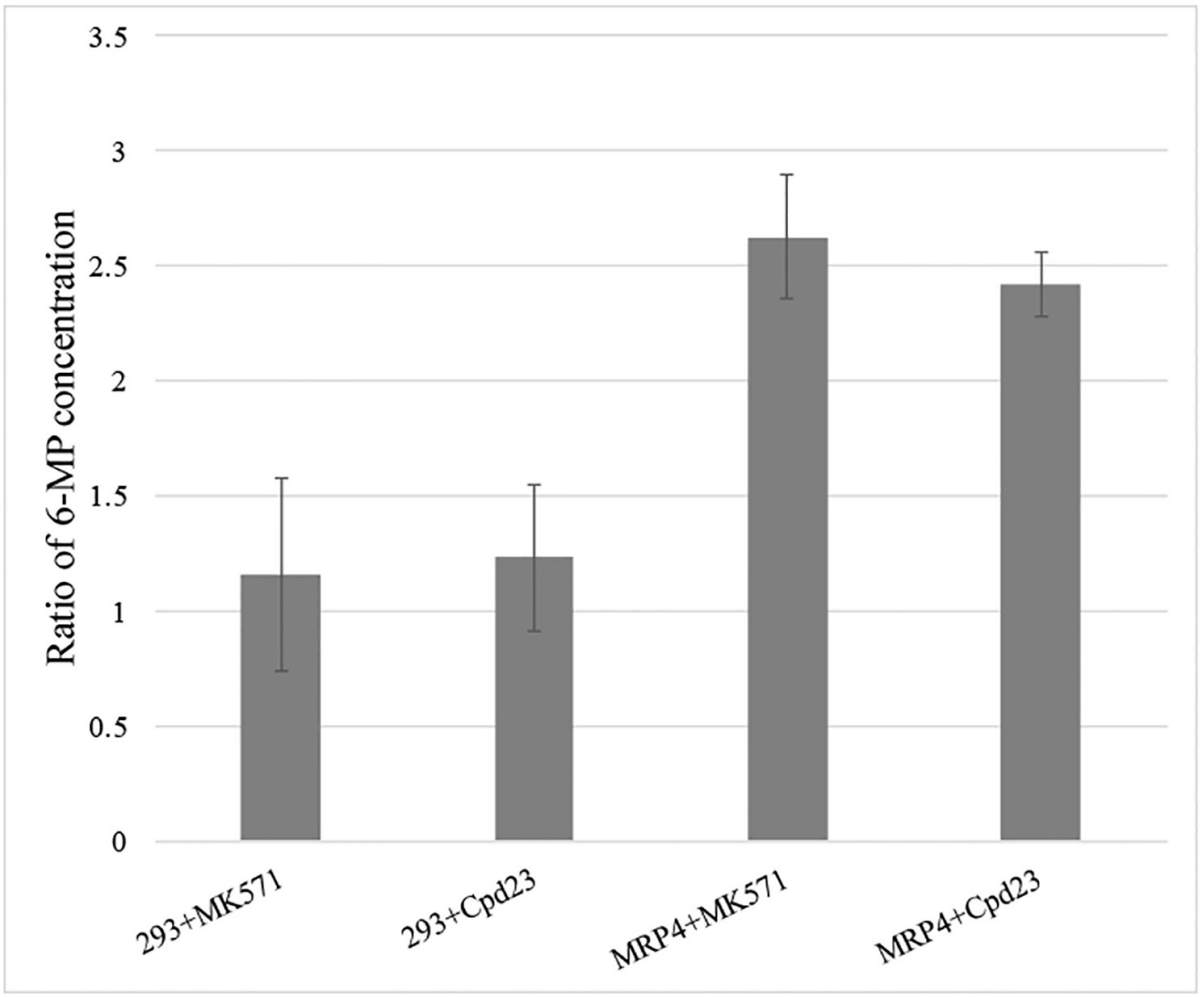
Effects of preincubation of HEK293 and HEK293/MRP4 cells with MK571 or Cpd23 at 50 μM on the accumulation of 6-MP. Y-axis represents the ratio of 6-MP concentration compared to only 6-MP treated HEK293 or HEK293/MRP4 cells, respectively. 293+MK571 and 293+Cpd23 stand for HEK293 cells with MK571 or **Cpd23**-treated, while MRP4+MK571 and MRP4+Cpd23 stand for HEK293/MRP4 cells with MK571 or **Cpd23**-treated, respectively. The results are the mean of independent experiments with standard deviation.

The accumulation of 6-MP in HEK293 and HEK293/MRP4 cells were examined. The intracellular accumulation of 6-MP in HEK293/MRP4 cells over 2 h was significantly lower than in HEK293. The effects of preincubation with 50 μM of MK571 or 50 μM of **Cpd23** on the accumulation of 6-MP in both HEK293 and HEK293/MRP4 cells are shown in Fig 8. Pretreatment of the HEK293/MRP4 cells with 50 μM MK571 for 2 h resulted in significantly increased accumulation of 6-MP by more than 1.5 fold. Preincubation of HEK293/MRP4 cells with **Cpd23** (50 μM) for 2 h also significantly increased the amount of 6-MP in cells, but the increased amplitude was slightly less than 1.5 fold. However, preincubation of either MK571 or **Cpd23** had little effect on 6-MP accumulation in HEK293 cells, which might explain the negligible effect of MK571 or **Cpd23** on the cytotoxicity of 6-MP in these cells. These findings also demonstrate that MRP4 does not participate in uptake of 6-MP in HEK293 cells because MK571 is a known inhibitor of MRP4.

**Fig 9:**
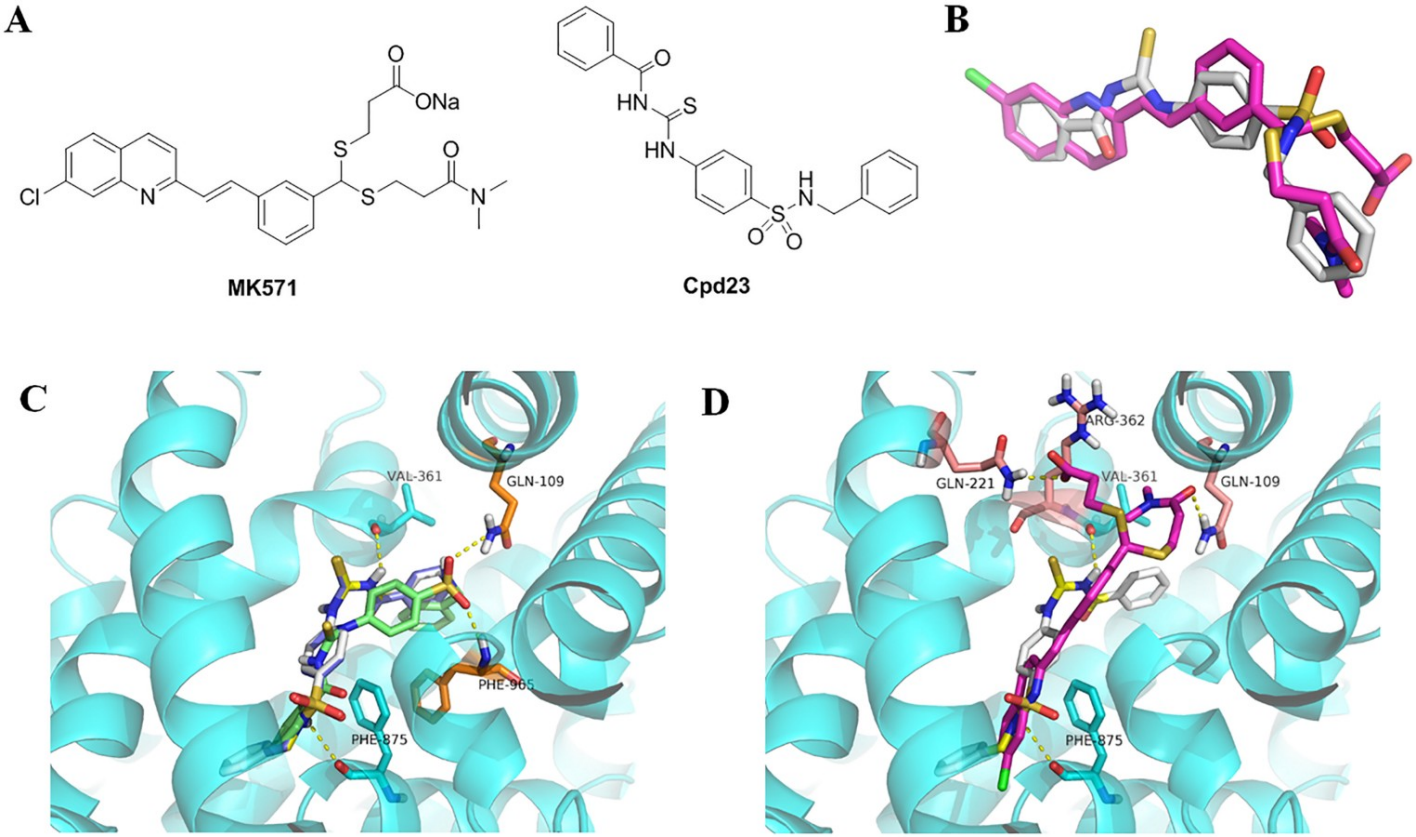
**A) Structures of MK571 and Cpd23. B) Three-dimensional alignment of MK571 and Cpd23 conformers (dock1). C) Interaction of docking poses of Cpd23 with the active site of the MRP4 model**. Key residues are shown in orange or cyan sticks, and the predicted hydrogen bonds are shown in yellow dashes. **D) Different binding modes of MK571 (dock1) and Cpd23 with the active site of MRP4**. Carbon atoms of MK571 and **Cpd23** are in magenta and white, respectively. Key residues are shown in salmon or cyan sticks, and the predicted hydrogen bonds are shown in yellow dashes.

In regard to the similarity to MK571, **Cpd23** is radically different with respect to their two-dimensional scaffold (Fig 9A), with very low ECFP_6 (0.096) and FCFP_6 (0.097) (S2 Table), but similar in 3D shape, with a Shape Tanimoto similarity value of 0.718 (compared to the dock1 query). Though there was no part in **Cpd23** aligned to the carboxyl group of MK571, the other three parts were comparable matched (Fig 9B). The benzene ring was not electrostatically similar to the amide group, but they could be well overlapped by shape. **Cpd23** had Shape Tanimoto values lower than 0.7 with the other two queries of MK571 (0.613 to dock2 and 0.648 to omega). If we did not use dock1 as one of these queries, we might not be able to acquire this compound with Shape Tanimoto cutoff of 0.7. Therefore, using more than one conformer as queries of 3D similarity search or adjusting the similarity cutoff would possibly improve the successful rate of identifying bioactive hits.

During the docking procedure, the three poses of **Cpd23** with the best predicted binding affinity were extracted and we also calculated the number of probable hydrogen bonds. The binding affinities were -9.7 kcal/mol, -9.7 kcal/mol and -9.8 kcal/mol, respectively, and every pose could form two potential hydrogen bonds with the receptor. The first two conformers were almost identical in conformation and might form hydrogen bonds with Val361 and Phe875, while the third one could probably form hydrogen bonds with Gln109 and Phe965 (Fig 9C). Compared to MK571, the binding modes of **Cpd23** are different and have interaction with diverse residues (Fig 9D). This might be because the active site is large and also MRP4 has large conformation changes during the substrate transport.

Most of the known MRP4 inhibitors contain a carboxylic acid group, while **Cpd23** is a non-carboxylic MRP4 inhibitor showing higher efficacy relative to MK571. Non-carboxylic MRP4 inhibitor might have better cell permeability, this should be confirmed in further study.

## Conclusions

In this study, a known MRP4 inhibitor, MK571, was used for ligand-based drug design, and a homology model of inward-facing MRP4 was used for structure-based drug design. The **Cpd23** was identified as a novel non-carboxylic MRP4 inhibitor from virtual screening of the SPECS database, showing equivalent activity to a higher concentration of MK571 in improving cell sensibility to anticancer drug 6-MP. The accumulation of 6-MP could increase to 2–3 times than those without **Cpd23**. To explore the structure and activity relationship and obtain more potent compounds, other analogs should be acquired by purchase or synthesis and evaluated biological activities in the future.

## Supporting information

S1 Table Computational results of 99 compounds. (XLSX)

S2 Table Information about seven selected compounds in SMILES format, calculated binding affinity results and 2D similarity to MK571. (XLSX)

S1 Fig Cell viability effect of Cpd23 on HEK293/MRP4 cells and Fa-CI plot of Cpd23 and 6-MP combined on HEK293/MRP4 cells. (PDF)

S2 Fig Table of contents graphic. Schema for virtual screening strategy, chemical structures of MK571 and Cpd23, as well as IC_50_ for 6-MP on HEK293 and HEK293/MRP4 cells independently or in the presence of MRP4 inhibitors.
(PDF)

## Abbreviations

6-MP: 6-Mercaptopurine
ABC: ATP binding cassette
cAMP: cyclic adenosine monophosphate
cGMP: cyclic guanosine monophosphate
CI: combination index
HTS: high-throughput screening
LTD_4_: Leukotriene D_4_
MDR: multidrug resistance
MRP: multidrug resistance protein
MTMA: membrane transport-modulating agents
NBD: nucleotide binding domain
PAINS: Pan Assay Interference compounds
TMD: transmembrane domain
TMH: transmembrane helix
VLS: virtual ligand screening
