# Supplementary material for: Discovery of novel multidrug resistance protein 4 (MRP4) inhibitors as active agents reducing resistance to anticancer drug 6-Mercaptopurine (6-MP) by structure and ligand-based virtual screening
Source: PLoS One. 2018 Oct 15;13(10):e0205175. doi: 10.1371/journal.pone.0205175 (PMC6188748; doi:10.1371/journal.pone.0205175)
Supplement: S1 Fig — (PDF) [file pone.0205175.s003.pdf]

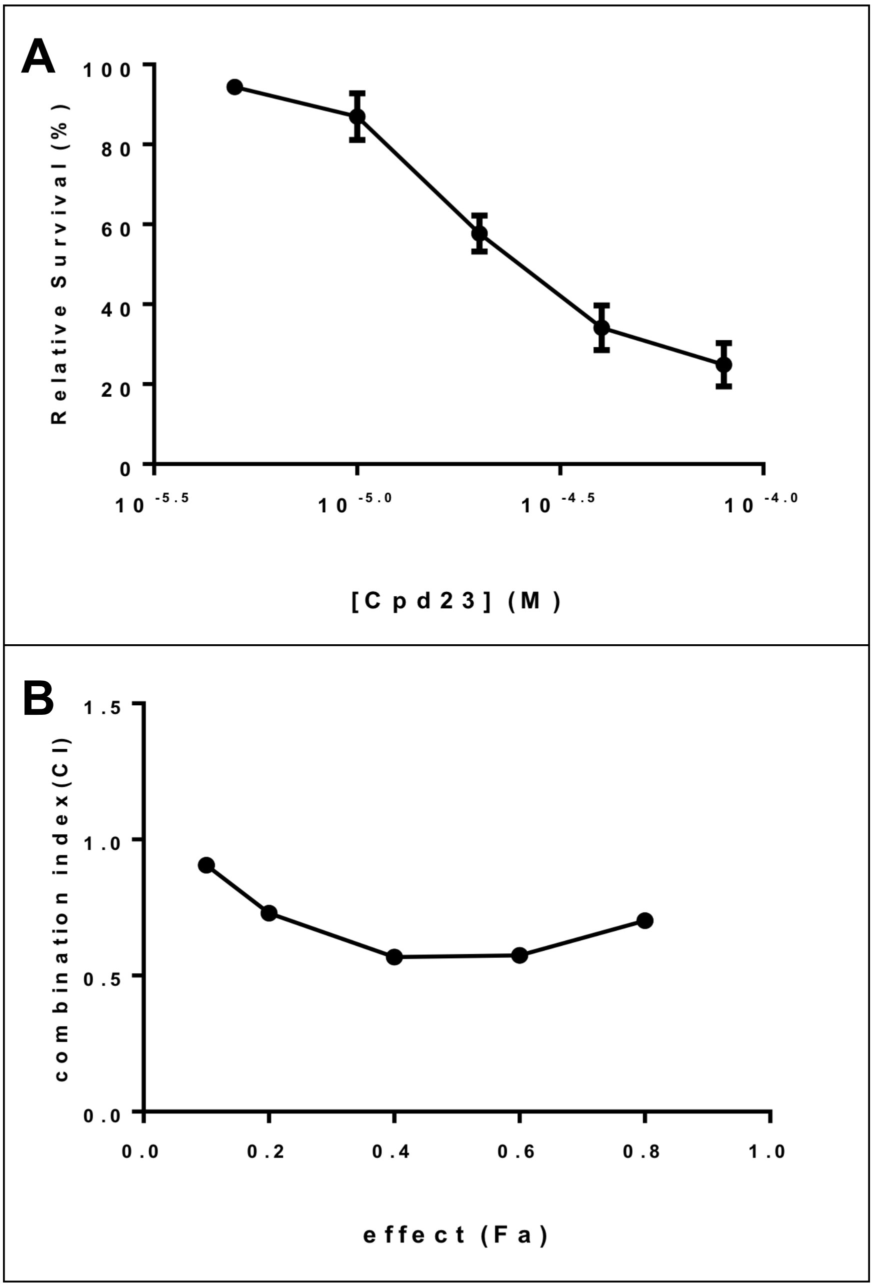

Figure S1. A) Cell viability effect of Cpd23 on HEK293/MRP4 cells and B) Fa-CI plot of Cpd23 and 6-MP combined on HEK293/MRP4 cells.
