# Supplementary figures and images for: Discovery of novel multidrug resistance protein 4 (MRP4) inhibitors as active agents reducing resistance to anticancer drug 6-Mercaptopurine (6-MP) by structure and ligand-based virtual screening
Source: PLoS One. 2018 Oct 15;13(10):e0205175. doi: 10.1371/journal.pone.0205175 (PMC6188748; doi:10.1371/journal.pone.0205175)

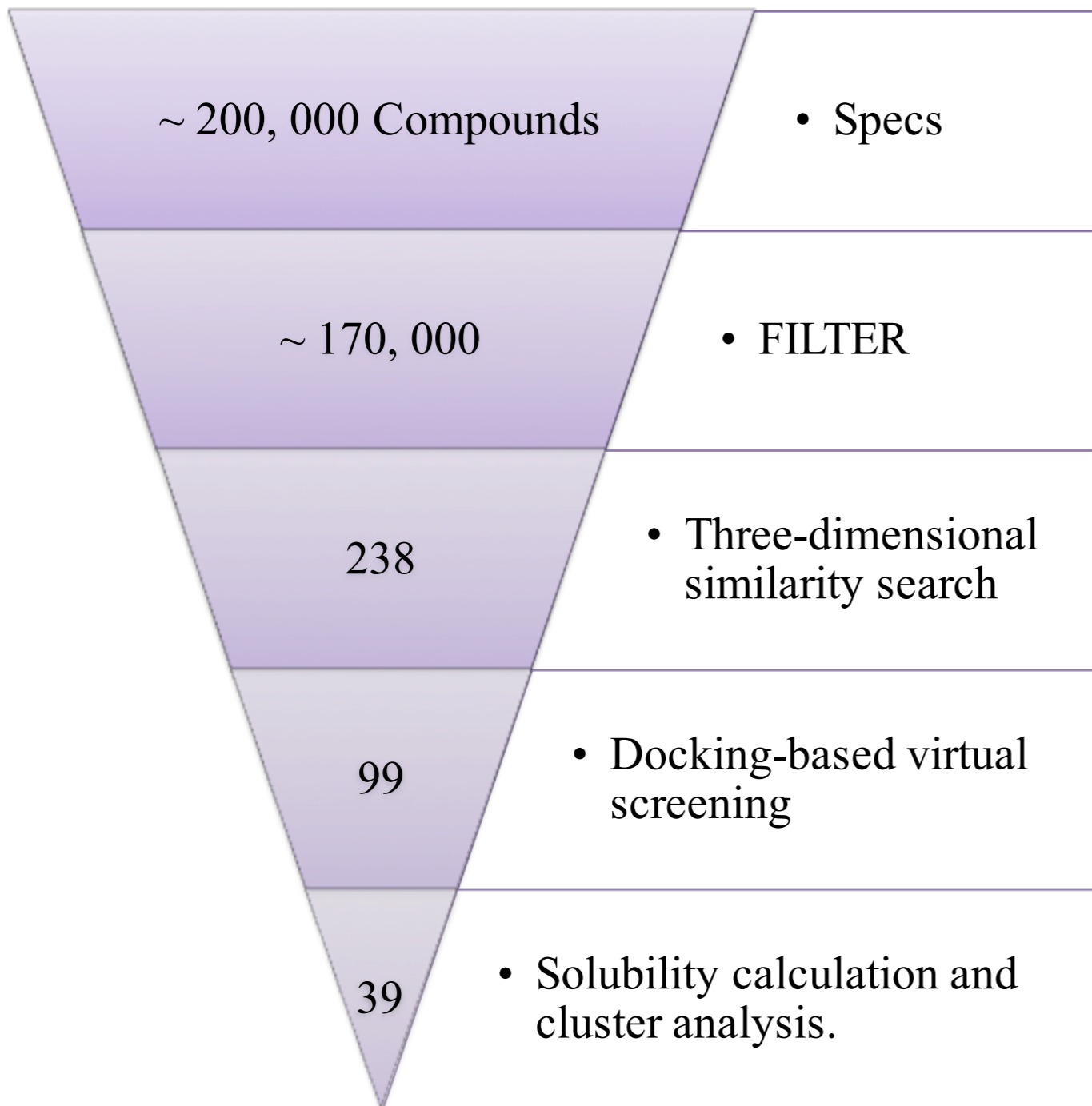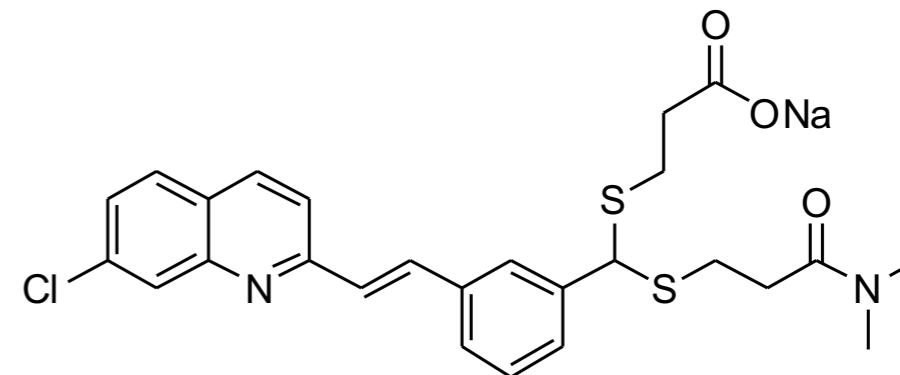

**MK571**

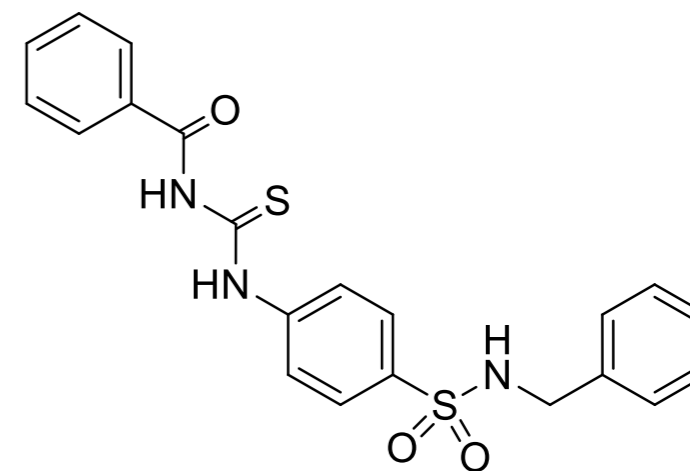

**Cpd23**

| Compound             | IC <sub>50</sub> (μM) |              |
|----------------------|-----------------------|--------------|
|                      | HEK293                | HEK293/MRP4  |
| 6-MP + DMSO          | 2.81 ± 0.35           | 14.11 ± 0.32 |
| 6-MP + MK571 (50 μM) | 2.66 ± 0.28           | 6.35 ± 0.68  |
| 6-MP + Cpd23 (5 μM)  | 1.84 ± 0.10           | 6.06 ± 0.81  |

Supplement: S2 Fig — Schema for virtual screening strategy, chemical structures of MK571 and Cpd23, as well as IC50 for 6-MP on HEK293 and HEK293/MRP4 cells independently or in the presence of MRP4 inhibitors. (PDF) [file pone.0205175.s004.pdf]
